# Bone Metastasis Phenotype and Growth Undergo Regulation by Micro-Environment Stimuli: Efficacy of Early Therapy with HGF or TGFβ1-Type I Receptor Blockade

**DOI:** 10.3390/ijms20102520

**Published:** 2019-05-22

**Authors:** Paola Bendinelli, Paola Maroni, Valentina Dall’Olio, Emanuela Matteucci, Maria Alfonsina Desiderio

**Affiliations:** 1 Dipartimento di Scienze Biomediche per la Salute, Molecular Pathology Laboratory, Università degli Studi di Milano, Via L. Mangiagalli 31, 20133 Milano, Italy; paola.bendinelli@unimi.it (P.B.); emanuela.matteucci@unimi.it (E.M.); 2 IRCCS Istituto Ortopedico Galeazzi, Via R. Galeazzi 4, 20161 Milano, Italy; paola.maroni@grupposandonato.it; 3 Cogentech S.c.a.r.l., Via Adamello 16, 20139 Milano, Italy; qPCR-service@cogentech.it

**Keywords:** mesenchymal epithelial transition, epithelial mesenchymal transition, hepatocyte growth factor, HGF, transforming growth factor β1, TGFβ1, Twist, Snail, HAI-1

## Abstract

Hepatocyte growth factor (HGF) and transforming growth factor β1 (TGFβ1) are biological stimuli of the micro-environment which affect bone metastasis phenotype through transcription factors, but their influence on the growth is scarcely known. In a xenograft model prepared with 1833 bone metastatic cells, derived from breast carcinoma cells, we evaluated mice survival and Twist and Snail expression and localization after competitive inhibition of HGF with NK4, or after blockade of TGFβ1-type I receptor (RI) with SB431542: in the latter condition HGF was also measured. To explain the in vivo data, in 1833 cells treated with SB431542 plus TGFβ1 we measured HGF formation and the transduction pathway involved. Altogether, HGF seemed relevant for bone-metastatic growth, being hampered by NK4 treatment, which decreased Twist more than Snail in the metastasis bulk. TGFβ1-RI blockade enhanced HGF in metastasis and adjacent bone marrow, while reducing prevalently Snail expression at the front and bulk of bone metastasis. The HGF accumulation in 1833 cells depended on an auxiliary signaling pathway, triggered by TGFβ1 under SB431542, which interfered in the transcription of HGF activator inhibitor type 1 (HAI-1) downstream of TGFβ-activated kinase 1 (TAK1): HGF stimulated Twist transactivation. In conclusion, the impairment of initial outgrowth with NK4 seemed therapeutically promising more than SB431542 chemotherapy; a functional correlation between Twist and Snail in bone metastasis seemed to be influenced by the biological stimuli of the micro-environment, and the targeting of these phenotype biomarkers might inhibit metastasis plasticity and colonization, even if it would be necessary to consider the changes of HGF levels in bone metastases undergoing TGFβ1-RI blockade.

## 1. Introduction

The bone marrow is the micro-environment of osseous metastases, and houses a heterogeneous population of cells and various matricellular proteins within the extracellular matrix, which represents a sponge for cytokines including growth factors, and confers mechanical stiffness [1,2,3,4]. Stroma-metastasis crosstalk occurs through paracrine stimulation and triggers a signaling network, variable over space and time. The biological stimuli are represented by hepatocyte (HGF), vascular endothelial (VEGF) and transforming β1 (TGFβ1) growth factors, Endothelin-1 and microRNA contained in exosomes [2,5,6,7,8,9]; the physical stimuli include proteins like protein acidic and rich in cysteine (SPARC), periostin, osteopontin as well as oxygen levels (from 1% to 6%) typical of hypoxic condition [2,10,11,12]. Thus, the plasticity of bone metastasis begins at the level of micro-environment, depends on the transcription factor profile, and consists in the switch from the mesenchymal phenotype of the initial steps to the epithelial phenotype, underlying the colonization of overt metastases [5,13,14,15,16]. Notably, in bone metastases HGF is mostly supplied by supportive cells, such as fibroblasts and megakaryocytes, through an exosome-mediated communication between stroma and metastatic cells [8,17].

Uncharacterized molecular mechanisms, behind the outgrowth of human skeleton metastasis from breast carcinoma, would be useful in personalized medicine as targets of therapy with benefits for bone metastatic treatment. The rationale of the present study was to show the relationship between bone metastasis growth and phenotype changes in response to growth factors. Thus, our specific aim was to evaluate whether the blockade of a key signaling pathway, such as that downstream of TGFβ1-type I receptor (RI) might induce alternative biological stimuli important for colonization of bone metastases. SB431542 chemotherapeutic agent inhibits TGFβ1-RI and undergoes preclinical studies [18]. In 1833 bone metastatic cells and in the 1833 xenograft model, we performed experiments under TGFβ1 plus SB431542 to clarify the interaction between TGFβ1 and HGF signaling pathways, and to evaluate HGF formation. For this, we assayed HGF activator inhibitor type 1/2 (HAI-1/2) critical for HGF formation, by regulating the pericellular activity of circulating hepatocyte growth factor activator (HGFA), and the cellular activity of type II transmembrane serine proteases (TTSPs) [19]. Novel therapies are devised to target the micro-environment [14], preventing the crosstalk with metastatic cells and the formation of osteoblastic/vascular niche(s) favorable for metastasis colonization. In this context, in the 1833 xenograft model of bone metastasis we verified the occurrence of a temporal hierarchy of HGF and TGFβ1-signaling pathways, with consequences on Twist and Snail distribution at the front and/or the bulk of bone metastases, likely influencing invasiveness and growth. 

Growth factors like TGFβ1 and HGF, and their associated transduction proteins, trigger epithelial-mesenchymal transition (EMT) and mesenchymal-epithelial transition (MET) programs in cancer cells by regulating the activity of Snail, Twist and ZEB [5,6,20,21]. TGFβ signaling pathway is active throughout the full course of bone metastasis [16]. HGF might influence a stem-like phenotype activating the Wnt pathway in 1833 bone metastatic cells, and in the 1833 xenograft model of bone metastasis [22,23]. Wnt activation is an early event in metastatic outgrowth [24]. Interestingly, HGF affects not only molecular events typical of EMT phenotype like stemness, anoikis resistance and survival, but also epithelial characteristics due to E-cadherin-dependent intercellular adhesion, which would favor the proliferation and drug responsiveness of metastatic cells [25,26]. Thus, the phenotype of human bone metastasis is intermediate/metastable with epigenetic control of key molecular players, such as HGF/Met receptor couple and E-cadherin, contributing to metastatic plasticity [5,8,13,17]. Previous studies suggest a mechanism by which HGF-dependent enhancement of TGFβ1 transactivation participates in the metastable phenotype of bone metastasis [5]. 

TGFβ binds to TGFβ1-type II receptor (RII) recruiting TGFβ1-RI, with consequent Smads 2/3 phosphorylation and formation of a complex with Smad4 (canonical pathway). Auxiliary pathway(s) may be also transduced by TGFβ through TGFβ-activated kinase 1 (TAK1), which activates c-Jun N-terminal kinase (JNK) and mitogen-activated protein kinase (p38) downstream, modulating biological responses like EMT, cell adhesion, migration and survival [27,28]. 

The micro-environment stimulus HGF was relevant for bone metastatic growth as suggested by the remarkable inhibition due to NK4, a competitive inhibitor of HGF [29], leading prevalently to Twist down-regulation in the metastasis bulk. In bone metastasis, HGF accumulated under SB431542 in the presence of TGFβ1, due to the impairment of HAI-1 transcription and of TAK1 phosphorylation upstream. Notably, TGFβ1-signaling blockade in vivo reduced Snail expression at the front and the bulk of bone metastasis, preventing the inhibitory effect of Snail on Twist transactivation. 

## 2. Results

### 2.1. The Blockade of Hepatocyte Growth Factor (HGF) or Transforming Growth Factor β1 (TGFβ1) Signaling Pathway Impaired Bone Metastasis Outgrowth and Prolonged Mice Survival 

The EMT and MET phenotypes of bone metastasis seem to be influenced by transcription factors under the biological stimuli of the micro-environment HGF and TGFβ1 [5,6,16], but the impact on colonization is scarcely known. HGF seems to affect epithelial characteristics [5,26], and TGFβ1 regulates the EMT program [3]. We clarified whether molecular mechanisms undergoing HGF or TGFβ1 regulation played a role in the growth of bone metastasis in a xenograft model prepared with 1833/TGL cells (luminescent), known to have a prevalent tropism for the skeleton [25].

**Figure 1:**
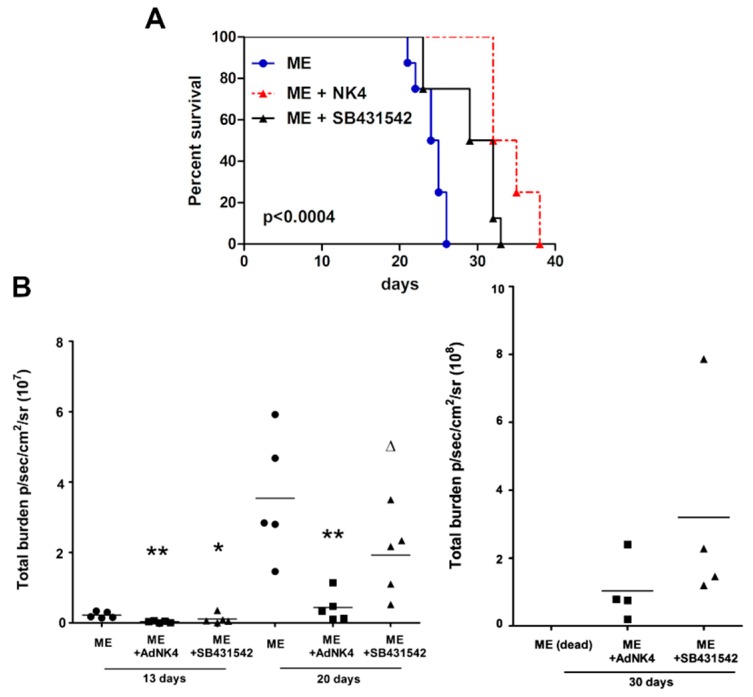
Effects of AdNK4 and SB431542 on animal survival and on bone metastasis outgrowth. (**A**) Survival curve on Kaplan–Meier plots of the data from mice exhibiting bone metastases (indicated as ME) exposed or not to AdNK4 or SB431542. The experiments started with 8 xenograft mice per group. (**B**) Changes of bioluminescence signals (total burden), corresponding to bone metastases of ME mice, treated or not with the inhibitors. The dot blots show the values of total burden for five mice at 13 and at 20 days, and for four mice at 30 days from 1833/TGL cell injection. At 30 days ME mice were dead. * *P* < 0.05, ** *P* < 0.005 versus ME value at the corresponding time; ^Δ^ *P* < 0.05 versus bioluminescence value under AdNK4 at 20 days.

Figure 1 reports survival data of experiments performed with NK4, expressed by an adenoviral vector (AdNK4), or with SB431542. NK4 prevents HGF binding to Met receptor and blocks HGF signaling; SB431542 inhibits the signaling pathway(s) downstream of TGFβ1-RI [18,29]. The Kaplan–Meier analysis showed that the blockade of the HGF/Met axis or TGFβ1 signaling prolonged mouse survival (Figure 1A). Mice exhibiting bone metastasis (indicated as ME) died within 22–26 days from the 1833/TGL cell injection, while mice treated with SB431542 and AdNK4 survived until 33 and 38 days, respectively. All the comparisons were made versus the ME group since the xenograft mice injected with the control adenoviral vector AdLacZ survived as ME [25]. The survival was more prolonged under NK4 than under SB431542.

Figure 1B shows the values of bioluminescence (total burden), corresponding to the comprehensive signals for bone metastasis colonization in the skull, chest, right and left hind limbs. Bioluminescence values decreased starting from 13 days after the two treatments in comparison with ME value. Of note, under AdNK4 86%–88% decreases occurred at 13 and 20 days, in respect to ME bioluminescence. Under SB431542, the total burden decreased by 62% at 13 days versus the ME bioluminescence value, but 20–30 days after SB431542 treatment the bioluminescent signal recovered over that of AdNK4 group. 

**Figure 2:**
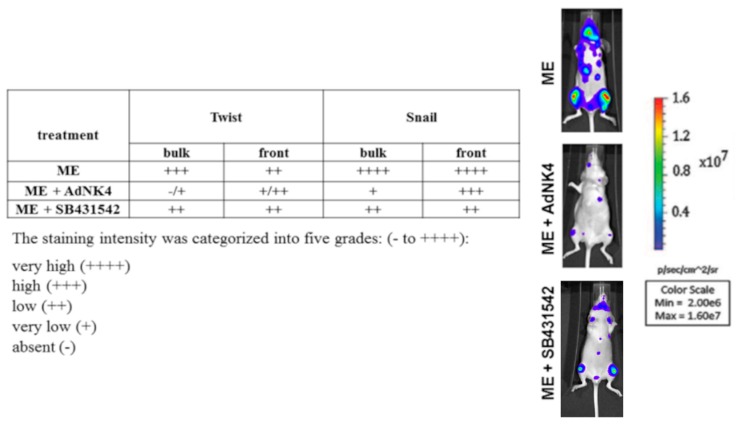
Semiquantitative evaluation of Twist and Snail expression. The expression of Twist and Snail was evaluated by immunohistochemistry on bone slides from 3 mice per group, i.e., ME at 25 days, and ME treated with AdNK4 or SB431542 at 32 days; the degree of positivity of the signals was shown as staining intensity. We report the bioluminescence images of representative mice from each of the three groups of treatments.

In further experiments, we investigated whether HGF and TGFβ1 differently influenced the expression, localization and function of transcription factors Twist and Snail in bone metastasis samples by immunohistochemistry using xenograft mice treated with AdNK4 or SB431542. Figure 2 reports the semiquantitative evaluation of Twist and Snail at the front and the bulk of bone metastasis. In bone metastases of untreated mice (ME), the expression of Twist and Snail was high (+++, bulk) and very high (++++, bulk/front), respectively. After AdNK4 exposure, Twist became absent/very low (−/+) in the bulk and low (+/++) in the front of bone metastasis. Notably, under AdNK4 the Snail signal remained high (+++) at the front opposite to the bulk (+). SB431542 reduced Twist and Snail to low values (++). 

Altogether, strong differences in the bioluminescence signals were observed at 20 days in the xenograft mice exposed to AdNK4 or SB431542 (Figure 2), in agreement with the quantitative data of Figure 1B, and confirming the remarkable efficacy of AdNK4 in slowing down bone metastasis growth. The images of the xenograft mice bioluminescence at various times under AdNK4 have been reported [25]. To explain the data obtained, in the following experiments we examined the possible interaction(s) between the growth factors HGF and TGFβ1 and between the transcription factors Twist and Snail.

### 2.2. Effects of the Blockade of HGF or TGFβ1 Signaling Pathway on Twist, Snail and HGF Expression in Xenograft Mice, and Regulation of Twist Transactivating Activity by Snail and HGF

We decided to verify whether the blockade of TGFβ1-RI might induce alternative biological stimuli, important for colonization of bone metastasis, affecting transcription factors downstream. Our present experiments with AdNK4 emphasized the critical role of HGF in bone metastasis growth. 

**Figure 3:**
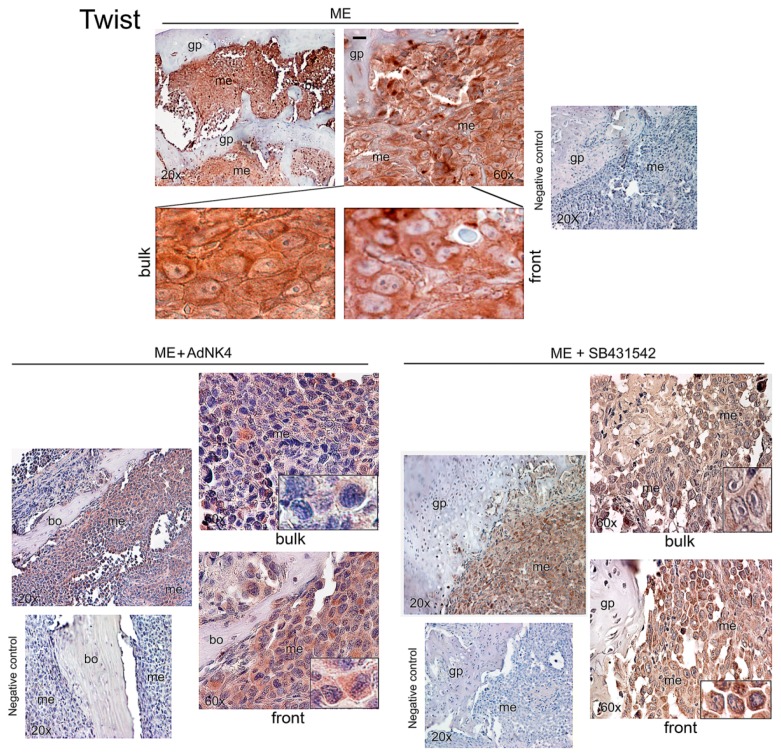
Effect of in vivo blockade of HGF or TGFβ1 signaling on Twist expression and localization in bone metastasis of the xenograft mice. Three animals were sacrificed at 25 days for ME, and at 32 days for ME+AdNK4 and ME+SB431542, and we show representative images for Twist immunostaining using bone slides of mouse 1; magnifications of details are reported in the insets. gp, growth plate; me, metastasis; bo, bone. Negative controls were performed without the specific antibody. Scale bar = 120 μm (reported in an exemplificative panel).

**Figure 4:**
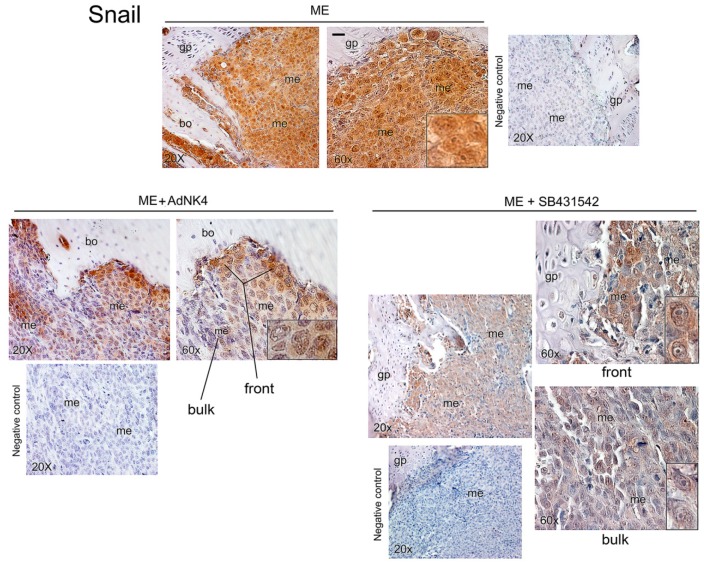
Effect of in vivo blockade of HGF or TGFβ1 signaling on Snail expression in bone metastasis of the xenograft mice. Three animals were sacrificed at 25 days for ME, and at 32 days for ME+AdNK4 and ME+SB431542, and we show representative images for Snail immunostaining using bone slides of mouse 1; magnifications of details are reported in the insets. gp, growth plate; me, metastasis; bo, bone. Negative controls were performed without the specific antibody. Scale bar = 120 μm (reported in an exemplificative panel).

**Figure 5:**
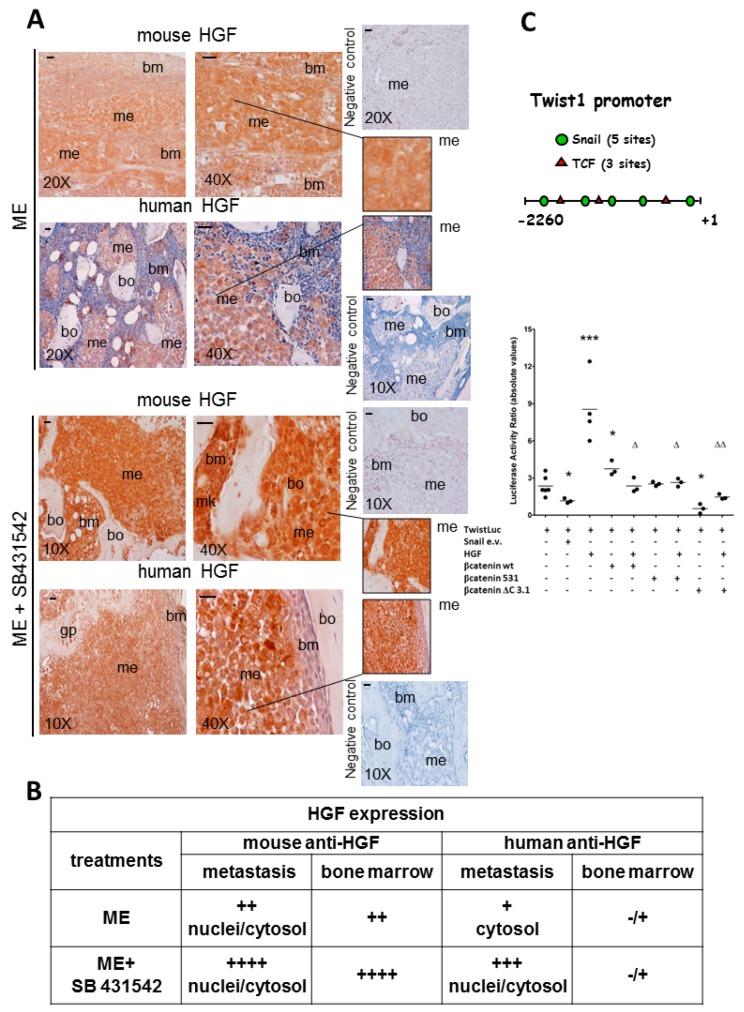
Enhancement of HGF expression in bone metastasis by SB431542: role played by HGF in Twist transactivation and interference of β-catenin. (**A**) Three animals were sacrificed at 25 days for ME, and at 32 days for ME+SB431542, and we show representative images for HGF immunostaining of bone slides from mouse 1, using anti-mouse and anti-human antibodies for HGF; magnifications of details are reported in the insets. gp, growth plate; me, metastasis; bm, bone marrow; mk, megakaryocytes; bo, bone. Negative controls were performed without the specific antibody. Scale bar = 120 μm. (**B**) In the table we report the semiquantitative evaluation of the degree of positivity of HGF signals at nuclear and cytosol levels for metastasis and bone marrow: the staining intensity with the two antibodies is shown. (**C**) Snail and TCF binding sites in Twist1 promoter are reported. The Twist1 gene reporter was transfected in 1833 cells, and Snail-expression vector co-transfection or HGF treatment was performed. Cells treated or not with HGF were transfected with various β-catenin expression vectors. The data of dot blots show the replicates: starvation (*n* = 6), HGF (*n* = 4), β-catenin expression vectors ± HGF and Snail expression vector (*n* = 3). * *P* < 0.05, *** *P* < 0.001 versus basal TwistLuc activity; ^Δ^ *P* < 0.05, ^ΔΔ^ *P* < 0.005 versus HGF-treated cells.

In Figure 3, Figure 4 and Figure 5 we report representative immunohistochemistry images of Twist, Snail and HGF in the bone metastasis of the xenograft mice, also after exposure to AdNK4 or SB431542. It is known that SB431542 blocks DNA methyltransferase1 [30], and that human HGF promoter shows four CpG islands (Gene Bank U370554) and mouse HGF promoter presents numerous methylation sites [8]. Figure 3 shows a high Twist signal in bone metastasis of ME mice, which was largely nuclear and perinuclear in the bulk, while in the metastasis front lining the bone/growth plate Twist was scarce in nuclei. AdNK4 abolished Twist expression in the nuclear compartment of bulk and invasive front, while a scarce Twist signal persisted in the cytosol of front metastatic cells. ME+SB431542 group showed lower Twist than ME mice all over bone metastasis. In conclusion, the nuclear Twist expression in the bulk was reduced by AdNK4 treatment much more than by TGFβ1-RI blockade. 

Snail was strongly expressed in bone metastasis of ME mice, both in nuclei and cytosol (Figure 4, inset). AdNK4 prevalently reduced Snail signal in the metastasis bulk, while being practically ineffective at the invasive front, as shown by the persistence of Snail positive cells facing the bone. Similarly, the invasive front facing the bone marrow was almost insensitive to AdNK4 exposure (Figure S1). SB431542 largely diminished Snail signal throughout bone metastasis (Figure 4), while enhancing HGF expression in metastasis and bone marrow (Figure 5A). Since the stimulatory effect of SB431542 on HGF expression was evident using mouse anti-HGF antibody, we suggest that HGF was produced by supportive cells of the bone marrow supplying the metastatic cells. Under SB431542, supportive cells like megakaryocytes (mk) seemed to produce HGF. Of note, stromal cells of metastasis micro-environment produce pro-HGF that is proteolitically cleaved in the mature form [19]. However, anti-human HGF antibody relieved HGF more in bone metastases of xenograft mice exposed to SB431542 than in those of untreated mice, probably due to HGF promoter demethylation under TGFβ1-RI blockade. For the first time we showed that HGF originated in bone metastases under chemotherapy (Figure 5A). 

The table shows the semiquantitative evaluation of HGF localized in bone metastasis and bone marrow (Figure 5B). In particular, for ME mice the signals were similar (++) in the two compartments using mouse anti-HGF antibody, while the HGF signal was practically absent in the bone marrow and was scarce in bone metastasis using human anti-HGF antibody. SB431542 enhanced HGF expression in nuclei and cytosol of bone metastasis using the two antibodies; in the bone marrow very high HGF signal was observed with anti-mouse antibody (++++) after SB431542 exposure.

To clarify the functional significance of HGF, the response of Twist activity to HGF and the correlation between Twist and Snail were evaluated (Figure 5C). Twist1 promoter presented five putative binding sites for Snail transcription factor, and this was the prerequisite to study Twist transactivation under Snail expression vector (e.v.). Snail e.v. down-regulated (−70%) the Twist luciferase activity, while Twist transactivation was enhanced (3.5-fold) by HGF (Figure 5C). In addition, Twist1 promoter showed three consensus sequences for T cell factor/lymphoid enhancer-binding factor (TCF/LEF). The β-catenin binding to TCF is critical for the activation of Wnt pathway [31]. To evaluate the influence of Wnt pathway on Twist activity, three different β-catenin expression vectors were tested. The experiments were performed with the β-catenin wild type (wt) form, the β-catenin constitutively active (531), i.e., a Ser37Cys mutant form not degradable, and the mutated β-catenin (ΔC3.1) lacking the C-terminal codons 696–781 [31]. The β-catenin wt enhanced (1.6-fold) Twist luciferase activity, while the mutated β-catenin (ΔC3.1) gave a strong inhibition (−80%); the three β-catenin constructs prevented the HGF-dependent enhancement of Twist transactivation (Figure 5C). Transfection efficiency of 1833 cells was 20%–25%, evaluated with β-galactosidase gene and colored reaction [32]. 

**Figure 6:**
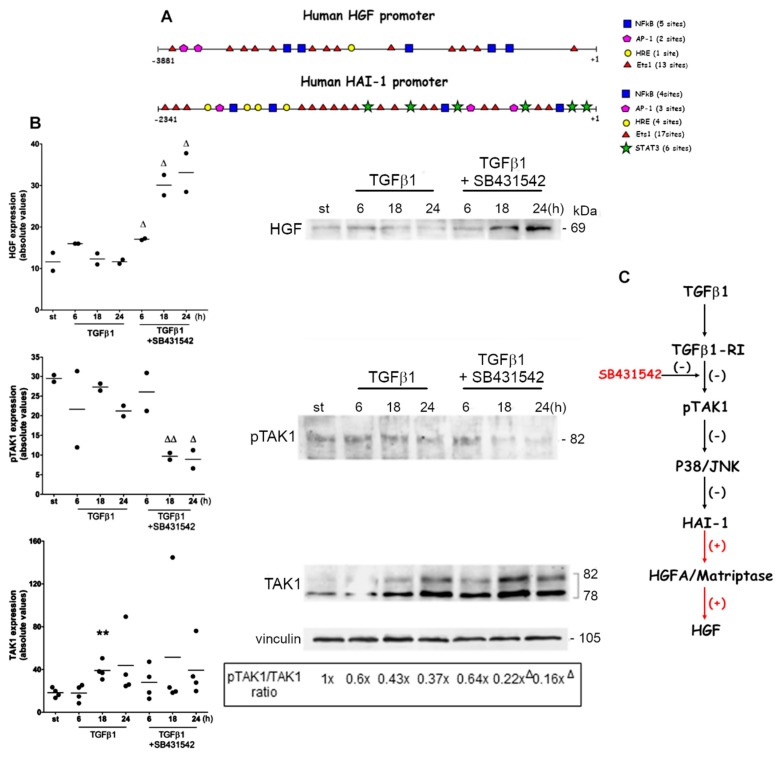
Effect of TGFβ1 exposure under TGFβ1-RI blockade on HGF formation. (**A**) Transcription factor binding sites in human HGF and HAI-1 promoters are reported. (**B**) Representative images of Western blots are shown; vinculin was used for normalization. The dot blots report the data of the Western blot experiments, which have been repeated two times for HGF and phosphoTAK1 immunoblotting, and four times for TAK1 immunoblotting. The pTAK1/TAK1 ratio was reported as fold-variations, that were calculated versus the starvation value. ** *P* < 0.005 versus the starvation value; ^Δ^ *P* < 0.05, ^ΔΔ^ *P* < 0.005 versus TGFβ1-treated cells at the corresponding time. (**C**) Schematic representation of the pathway leading to HGF formation under SB431542 treatment of TGFβ1-exposed 1833 cells. (−) Pathway inhibition; (+) pathway activation.

Altogether, SB431542 was more effective than AdNK4 in diminishing Snail localized at the bulk and the front of bone metastases, indicating that Snail was regulated by TGFβ1 signaling. Of note, the TGFβ1-RI blockade increased HGF in bone metastases. The HGF produced under chemotherapy with SB431542 would regulate Twist activity, and β-catenin/TCF transcription factor might interfere in Twist function. In this experimental condition with SB431542, changes of epigenetic regulatory mechanisms seemed to be responsible for the HGF increase in bone metastases, even if the triggering of an auxiliary pathway controlled by TGFβ1 cannot be excluded, and the data at this regard are reported in the following Figure 6. 

### 2.3. Auxiliary Pathway Responsible for HGF Accumulation under TGFβ1-RI Blockade 

Due to the importance of explaining the in vivo data, i.e., the short-term efficacy of chemotherapy against TGFβ1-RI, and the concomitant HGF formation, we evaluated the signaling events triggered by SB431542 in the presence of TGFβ1 in 1833 cells.

First, we examined the promoters of HGF and HAI-1: the presence of various consensus sequences indicated the cooperation of different transcription factors for gene expression (Figure 6A). Often genes that work in tandem undergo similar regulation, and this might be the case of HGF and HAI-1, inhibitors of pro-HGF activation [19]. Of note, only the promoter of HAI-1 shows six consensus sequences for signal transducers and activators of transcription (STAT)3, which might be implicated in NF-*k*B-dependent activation of the Janus kinase (JAK)/STAT signaling pathway [33], due to a crosstalk between p38/JNK and STAT3 [34]. In a non-canonical pathway triggered by TGFβ1, the TAK1 initiates signaling events comprising p38/JNK/STAT3 activation [27]. Based on these data, we hypothesize that STAT3 might intervene in HAI-1 regulation downstream of TGFβ1/TAK1. Then, Western blot experiments were performed to evaluate TAK1 and its phosphorylation, and HGF levels under exposure to TGFβ1 alone and to its combination with the chemotherapy drug SB431542, to block the type I receptor (Figure 6B). In 1833 cells exposed to TGFβ1 plus SB431542, the expression of HGF augmented during all the observation period, while TAK1 phosphorylation decreased between 18 and 24 h; TGFβ1 enhanced TAK1 protein level at 18 h. The phosphoTAK1/TAK1 ratio was down-regulated by the exposure to TGFβ1 plus SB431542 at 18 and 24 h, in respect to TGFβ1 alone at these times. Also, at 24 h HAI-1 transcript levels were analyzed. HAI-1 transcription showed a 1.53 ± 0.06 fold-increase under TGFβ1 (*P* < 0.05) or a 2.10 ± 0.04 fold-increase under SB431542 (*P* < 0.005), in respect to starvation value. Under the combination of TGFβ1 plus SB431542, HAI-1 mRNA level was 40% lower versus SB431542 (*P* = 0.001) and 20% lower versus TGFβ1 (*P* = 0.07), while being unchanged (1.24 ± 0.09 fold) in respect to starvation value. HAI-2 was unmodified by the treatments. It is known that HAI-1 and 2 are differently regulated in cancer [19].

Normally, the HGF activation from pro-HGF via HGFA and TTSP Matriptase, and the inhibition of the HGF production by HAI-1 take place at the same time. A regulatory loop for HAI-1 expression is controlled by HGF via NF*k*B/STAT3 [35,36]. As suggested in Figure 6C, the blockade of type I receptor in the presence of TGFβ1 led to the decrease of TAK1 phosphorylation and HGF accumulation, which might depend on the inhibition of p38/JNK/STAT3 and HAI-1. Consequently, in 1833 cells the observed increase in HGF was probably due to pro-HGF activating proteases like HGFA/Matriptase.

## 3. Discussion

We give insights into the roles of HGF and TGFβ1 in determining Twist and Snail profile at the center and invasive front of bone metastasis from breast carcinoma. The molecular changes occurring in the bulk and front of bone metastasis might be relevant for colonization due to MET phenotype. The relationship between HGF- and TGFβ1-signaling pathways seemed to be important for metastasis plasticity and resistance to therapy. NK4 which competitively blocks HGF binding to the Met receptor, delayed bone metastasis growth for a long period in the xenograft model as compared to SB431542, which increased HGF driving the recovery of metastasis colonization in the skeleton. 

The hierarchical involvement of growth factors might be important for the differential functions of Twist and Snail in the epithelial-like bulk versus the mesenchymal leading edge of osteolytic metastasis, and for the dynamic phenotype related also to the function of HAI-1. Of note, HAI-1 was evaluated in bone metastatic 1833 cells, which showed fairly elevated HAI-1 expression at a difference with various types of cancer [19]. HAI-1 function might be related to the presence of junctional proteins [19], and to the epithelial phenotype beyond the formation of HGF in bone metastases. Under SB431542 plus TGFβ1, the HAI-1 transcript was lower than under the single therapeutic treatment, in agreement with HGF accumulation. HGF is synthesized as a precursor, and Kallikrein 14 at low concentration cleaves pro-HGF into its two-chain active form, but it is also involved in the degradation of HAI-1 [37]. HAI-1 inhibits the pro-HGF activators HGFA (soluble) and the TTSPs Matriptase and hepsin (transmembrane) [19].

HGF played a key role in the expression of nuclear and cytosolic Twist in the bone metastasis bulk, because NK4 practically abolished the Twist signal, consistent with the function of HGF-dependent Twist expression in the establishment of skeleton metastases and with the control of epithelial-like phenotype by HGF [5]. In contrast, at the invasive front Snail expression was almost irresponsive to NK4: this finding indicated the EMT phenotype of metastatic cells at the interface with the bone and bone marrow of osteolytic metastasis. Altogether, a new spatio-temporal regulation of EMT/MET was shown in bone metastases, originating from breast carcinoma, consistent with metastases from colon and squamous cell carcinomas [38,39].

TGFβ1 activating JNK phosphorylates Ser68 of Twist important for the stabilization, but not for the nuclear localization [40]. TGFβ1-RI blockade down-regulated both Twist and Snail expression throughout bone metastasis, while increasing HGF formation due to an auxiliary pathway triggered by TGFβ1. The explanation from a mechanistic point of view might be that the reduction of TAK1 phosphorylation, likely affecting p38/JNK downstream, prevented HAI-1 transcription via STAT3 under the treatment with SB431542 in the presence of TGFβ1, in respect to SB431542 alone. We suggest that the impairment of TGFβ1-RI favored HGF formation from the cleavage of pro-HGF, which is furnished by the supportive cells of the osseus micro-environment, such as fibroblasts and magakaryocytes [12,25]. This is the reason why HGF expression was shown with anti-mouse HGF antibody in the bone metastases of xenograft mice. Notably, bone metastatic cells might express HGF, as relieved with anti-human HGF antibody, probably due to the epigenetic changes occurring under SB431542 [30].

HGF of micro-environment origin is supplemented to bone metastatic cells by exosomes [8]. Also, exosomes are present in fetal bovine serum [41], and they might supply growth factors such as HGF to 1833 cells cultured under SB431542 plus TGFβ1. 

The regional distribution of the transcription factors Twist and Snail influenced the bone metastatic phenotype, i.e., EMT at the invasive front regulated by TGFβ1-dependent Snail activity, and MET in the bulk of bone metastasis implicating HGF via Twist1 phosphorylation (Figure S2). HGF as well as matrix stiffness [42] might drive nuclear translocation of phosphoTwist1, which is important for the transcription factor activation mediated by Wwox [5]. However, the NK4-dependent blockade of the HGF/Met axis might affect also Snail expression, but limited at the bulk, by impairing HIF-1α expression and HIF-1 activity which regulates Snail [15,43]. 

**Figure 7:**
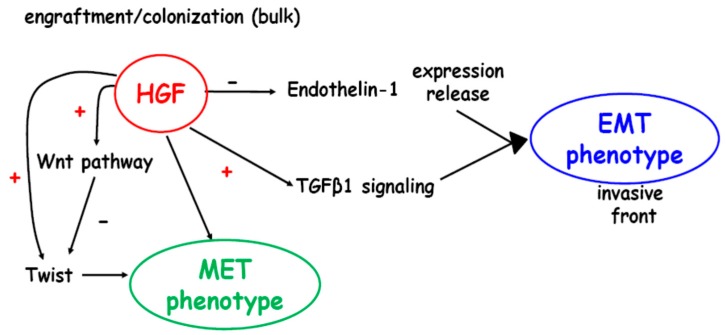
Bone metastases from breast carcinoma show a different phenotype at the invasive front (epithelial-mesenchymal transition, EMT) and in the bulk (mesenchymal-epithelial transition, MET), and the underlying molecular profiles regulated by HGF and TGFβ1 are schematized. (−) Pathway inhibition; (+) pathway activation.

A schematic representation of bone metastasis plasticity is reported (Figure 7). The invading metastatic cells, showing mesenchymal characteristics at the time of arrest in the bone, were likely to switch to the epithelial phenotype for successful outgrowth; a reversion to EMT might occur after colonization. The phenotypic changes would be related to proliferation rate and chemotherapy resistance of bone metastasis: the MET phenotype seems more amenable to treatment with classical and new therapies [44]. 

TGFβ1 produced by stromal cells influences carcinogenesis in adjacent epithelia through HGF signaling, important for epithelial proliferation [45]. HGF indirectly affects the invasive front down-regulating Endothelin-1 expression/release, important for E-cadherin expression [23]. 

HGF of the micro-environment mediates resistance to several targeted therapeutic agents [46], and the HGF blockade would be responsible for chemoresistance reduction through down-regulation of Twist, the activator of an osteoblastic program related to bone tropism [47]. As shown here, HGF might drive the resistance to the anti-TGFβ1-RI therapy because of an auto-amplifying regulatory loop, dependent on HGF, which regulates the TGFβ1 transactivation [5]. TGFβ1 exerts immunosuppressive, proangiogenic and EMT inducer roles [48,49], with an increase of Snail expression responsible for the inhibition of Twist transactivating activity. The Wnt pathway seemed to exert a partial competition on HGF-triggered Twist1 transactivation/expression (Figure 7). 

## 4. Materials and Methods 

### 4.1. Reagents and Plasmids

SB431542 (4-[4-(1,3-benzodioxol-5-yl)-5-(2-pyridinyl)-1H-imidazol-2-yl]-benzamide hydrate) was from Cayman Chemical (Ann Arbor, MI, USA). The adenoviral vectors expressing NK4 (AdNK4) and the control AdLacZ were furnished by Dr. T. Nakamura (Osaka University, Osaka, Japan). Recombinant human HGF and TGFβ1, and the anti-mouse HGF antibody were from R&D System (Abingdon, UK). Anti-Twist 1/2 (H-81) antibody was from Santa Cruz-Biotechnology (Santa Cruz, CA, USA); anti-phosphoTwist1 (Ser42) antibody was a kind gift of Dr. B. Hemmings (Friedrich Mieschern Institute for Biomedical Research, Basel, Switzerland); anti-Snail 1/2 (ab53519) antibody was from Abcam (Cambridge, UK); anti-human HGFα (H487) antibody was from Immuno-Biological Laboratories Inc. (Minneapolis, MN, USA); anti-TAK1, anti-phosphoTAK1 (Thr184/187) and anti-vinculin antibodies were from Cell Signaling Technology (Beverly, MA, USA). The construct containing the Twist promoter (TwistLuc) was from Dr. M.-C. Hung (University of Texas, TX, USA); the Snail expression vector was from Dr. J. I. Yook (Yonsei University, Seoul, Republic of Korea) and the β-catenin expression vector was from Dr. O. Maeda (Nagoya University, Nagoya, Japan). 

### 4.2. Xenograft Model Preparation

1833 cells were derived from MDA-MB231 breast carcinoma cells, and were retrovirally transfected with HSV1-tk/GFP/firefly luciferase (1833/TGL) in the laboratory of Dr. J. Massagué (Memorial Sloan-Kettering Cancer Center, New York, NY, USA) [50]. We injected 1833/TGL cells (5 × 10^5^) into the hearts of nu/nu mice, and the animals were divided into four groups: mice injected with 1833/TGL (ME, *n* = 8); ME treated with SB431542 (ME+SB431542, *n* = 8); ME treated with AdNK4 (ME+AdNK4, *n* = 8) and ME injected with AdLacZ (ME+AdLacZ, *n* = 3). Each mouse of AdNK4 group received i.v. 10^9^ pfu of the adenoviral vector expressing NK4 every 5 days, and AdLacZ was administered in the corresponding control group; we used a schedule with multiple AdNK4 injections to maintain elevated circulating NK4 levels, permitting NK4 access to the bone marrow [25]. The protocol for preparation of AdNK4 and AdLacZ adenoviral vectors has been reported [25]. SB431542 was administered i.p. at the dose of 0.2 mg/mouse, 5 times a week [51]. Animal studies were performed in conformity with the institutional guidelines, in compliance with national and international laws and policies (Directive 2010/63/EU); mice were suppressed to prevent suffering. Bone metastasis growth was monitored as bioluminescence using Xenogen IVIS 200 System (Caliper Life Sciences, Hopkinton, MA, USA) at Transgenic-Operative Products s.r.l. (Lodi, Italy) [25]. 

### 4.3. Immunohistochemistry Assay

The bones were fixed in 10% neutral buffered formalin, decalcified in Mielodec (Bio-Optica, Milan, Italy) and embedded in paraffin; serial sections (4 μm) for each specimen from three mice per group were stained with hematoxylin and eosin to verify bone metastasis presence. After antigen retrieval (95 °C for 20 min at pH 6 in antigen-unmasking solution, Vector Laboratories, Burlingame, CA, USA), sections were treated for 10 min with 0.1% (*v/v*) H_2_O_2_ and blocked with normal serum. Immunostaining was performed on bone specimen slices from three mice with anti-Twist (1:200), anti-Snail (1 µg/mL), anti-human HGF (3 μg/mL) or anti-mouse HGF (10 μg/mL) antibody, using a streptavidin-biotin system (ABC kit, Santa-Cruz Biotechnology) and diaminobenzidine substrate, and counterstaining with Meyer’s haematoxylin [25]. Negative-control sections were subjected to the same staining procedure without the primary antibody.

### 4.4. Cell Cultures and Treatments 

The 1833 cells were routinely maintained in Dulbecco’s modified Eagle’s medium (DMEM) containing 10% (*v/v*) fetal bovine serum (FBS), and were used after 2 or 3 passages in culture. Cultured cells under starvation were treated with HGF (100 ng/mL) or with TGFβ1 (5 ng/mL), which was used alone or in combination with 5 µM SB431542. The addition of SB431542 to the cultured cells was performed 1 h before TGFβ1 [5,52,53]. We authenticated 1833 cells with the method of short-tandem repeat profiling (STR) of nine highly polymorphic STR loci plus amelogenin on September 2014 (Cell Service from IRCCS-Azienda Ospedaliera Universitaria San Martino-IST-Istituto Nazionale per la Ricerca sul Cancro, Genova, Italy). The number of replicates for each type of experiment is reported in the legends of the Figures.

### 4.5. Western Blot Analysis

Total (100 µg) and nuclear (50 µg) protein extracts from treated cells were examined by Western blot; immunoblots were performed with anti-human HGF (3 μg/mL), anti-TAK1 (1:1000), anti-phosphoTAK1 (1:1000), anti-Twist1 (1:200), anti-phosphoTwist1 (1 μg/mL) and anti-vinculin (1 μg/mL). Densitometric analysis was performed after reaction with enhanced chemiluminescence (ECL) plus kit from Thermo-Fisher Scientific (Waltham, MA, USA) [5].

### 4.6. Quantitative Reverse-Transcription Polymerase Chain Reaction (RT-PCR) Detection of mRNAs 

Total RNA was extracted from treated 1833 cells using Pure Link RNA mini kit (Invitrogen, Monza, Italy). The RNA was quantified using “NanoDrop”, and the quality verified running 500 ng of RNA on a 1% Agarose gel. 1 μg of RNA was retrotranscribed using Quanta kit (VWR International, Milano, Italy). For two samples, RT minus was made to check the absence of genomic DNA. For gene expression analysis, samples of cDNA (5 ng) were amplified (in triplicate) using TaqMan Gene Expression Assay (HAI-1/SPINT1 assay ID: Hs00173678_m1; HAI-2/SPINT2 assay ID: Hs01070442_m1; Glyceraldehyde-3-phosphate dehydrogenase (GAPDH) assay ID: Hs99999905_m1, Thermo-Fisher Scientific). Real-time polymerase chain reaction (PCR) was carried out on the 7900HT Fast Real-Time PCR (Thermo-Fisher Scientific), using a pre-PCR step of 20s at 95 °C, followed by 40 cycles of 1s at 95 °C and 20s at 60 °C. Samples were amplified with primers and probes for each target gene, and for all these genes one no template control (NTC) sample was run. Raw data (CT) were analyzed with “Biogazelle qbase plus” software, and the fold changes (versus starvation value) were expressed as CNRQ (calibrated normalized relative quantity) of the means with Standard Error (S.E.). GAPDH was used as housekeeping gene. The starvation values reported as 2^−ΔΔCT^ were 3.37 × 10^−3^ for HAI-1 and 230.53 × 10^−3^ for HAI-2. The entire process (extraction, retrotranscription, gene expression and data analysis) was performed by the qPCR-Service at Cogentech (Milano, Italy).

### 4.7. Transient Transfection and Luciferase Reporter Assay 

The 1833 cells in 24-multiwell plates at 70–80% of confluence, were incubated with 0.4 µg/mL of TwistLuc gene reporter using a mixture (3:1) of DNA:Fugene 6 (Roche Applied Science, Monza, Italy), which contained the internal control pRL-TK (*Renilla* luciferase plasmid). These cells were exposed to HGF (200 ng/mL), or were co-transfected for 24 h with expression vectors (0.4 µg/mL), or equivalent amounts of empty vectors. The absolute values were calculated by the software, and corresponded to Firefly/*Renilla* luciferase activity ratios [5]. 

### 4.8. Statistical Analysis

The survival data of the xenograft mice were analyzed by the Kaplan–Meier method and the Log-rank (Mantel–Cox) test. *P* < 0.05 was considered significant. The values of luciferase activity, Western blot densitometry and PCR assay were analyzed using Student’s *t*-test, with *P* < 0.05 considered significant. 

## 5. Conclusions

The molecular network influencing the phenotype of bone metastasis from breast carcinoma depends on the variability of cellular context and the micro-environment signals, as well as tissue heterogeneity. Inhibition of metastatic growth is complicated by the plasticity of cancer cell behaviors, and the evolving nature of the micro-environment.

The knowledge of the influence of micro-environment on transcription factors controlling EMT/MET and HAI-1 expression, seems important to devise therapies affecting the metastasis phenotype. The present findings indicate that the hierarchy of HGF and TGFβ1 was important for colonization, as a consequence of phenotype changes due to the transcription factors Twist and Snail in the bulk (NK4/SB431542 targets) and Snail in the invasive front (SB431542 target). It was noteworthy that the therapeutic blockade of TGFβ1 signaling downstream of TGFβ1-RI, increased HGF expression, with possible consequences on angiogenesis and/or anoikis of bone metastases, suggesting that different micro-environment stimuli have to be concomitantly blocked to prevent metastasis outgrowth.

## Supplementary Materials

Supplementary Materials can be found at https://www.mdpi.com/1422-0067/20/10/2520/s1: Figure S1: Pattern of Snail expression in bone metastases under AdNK4; Figure S2: Evaluation of Twist1 and phosphoTwist1 expression in nuclei of 1833 cells treated with HGF.

## Abbreviations

EMT	Epithelial-mesenchymal transition
MET	Mesenchymal-epithelial transition
TGFβ1	transforming growth factor β1
HGF	hepatocyte growth factor
VEGF	vascular endothelial growth factor
TGFβ1-RI	TGFβ1 type I receptor
TAK1	TGFβ-activated kinase1
JNK	c-Jun N-terminal kinase
HAI-1	HGF activator inhibitor-1
HAI-2	HGF activator inhibitor-2
