# Supplementary material for: Bone Metastasis Phenotype and Growth Undergo Regulation by Micro-Environment Stimuli: Efficacy of Early Therapy with HGF or TGFβ1-Type I Receptor Blockade
Source: Int J Mol Sci. 2019 May 22;20(10):2520. doi: 10.3390/ijms20102520 (PMC6567054; doi:10.3390/ijms20102520)
Supplement: Supplementary file 1 [file ijms-20-02520-s001.pdf]

SUPPLEMENTARY MATERIAL

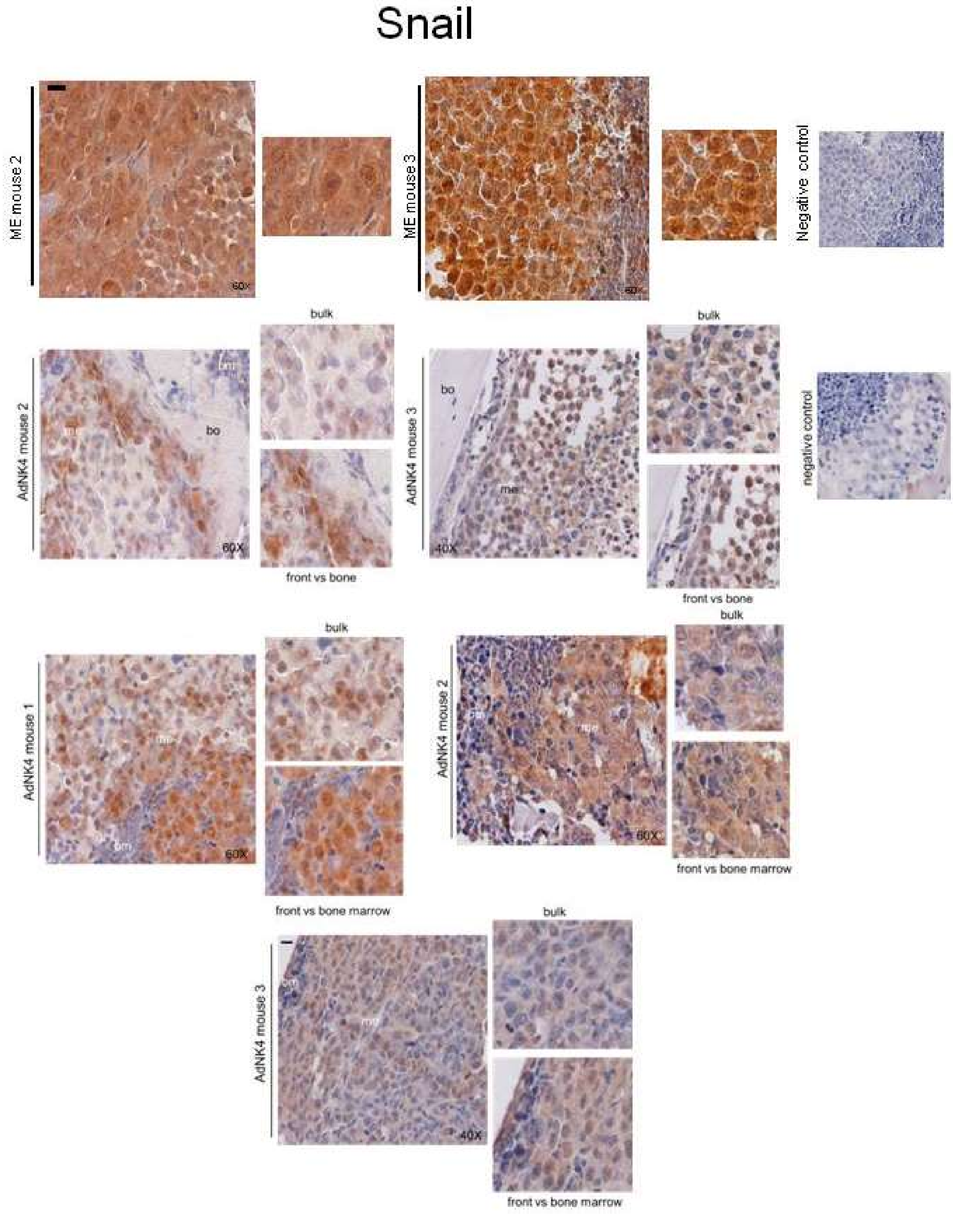

Supplementary Figure 1. Pattern of Snail expression in bone metastases under AdNK4. Groups of three xenograft mice were treated or not with AdNK4 to block HGF/Met receptor axis, and bone metastasis bearing mice (ME, taken as controls) were sacrificed at 25 days, while ME+AdNK4 group of mice was sacrificed at 32 days. Slides from bone samples were used to examine Snail expression by immunohistochemistry, and representative images are reported. Magnification is shown in the insets. In particular, under AdNK4 we examined the distribution of Snail signal in the bulk, and in the front towards bone and the bone marrow. To extend the data of mouse 1 shown in Figure 3 of the paper, for ME and ME+AdNK4 front vs. bone we show the images for mice 2 and 3; for AdNK4 effect on Snail in the front vs. bone marrow we show images for mice 1-3. Altogether, the data show a very high Snail signal in bone metastases of ME. Under AdNK4 the Snail expression almost disappeared from the bulk while at the fronts towards bone and bone marrow the signals persisted in great extent. This indicated that the invasive fronts showed a mesenchymal phenotype largely insensitive to HGF/Met receptor blockade, since HGF was likely to influence also epithelial characteristics of bone metastases. me, bone metastasis; bm, bone marrow; bo, bone. Negative controls were performed without specific antibody. Scale bar=120 μm.

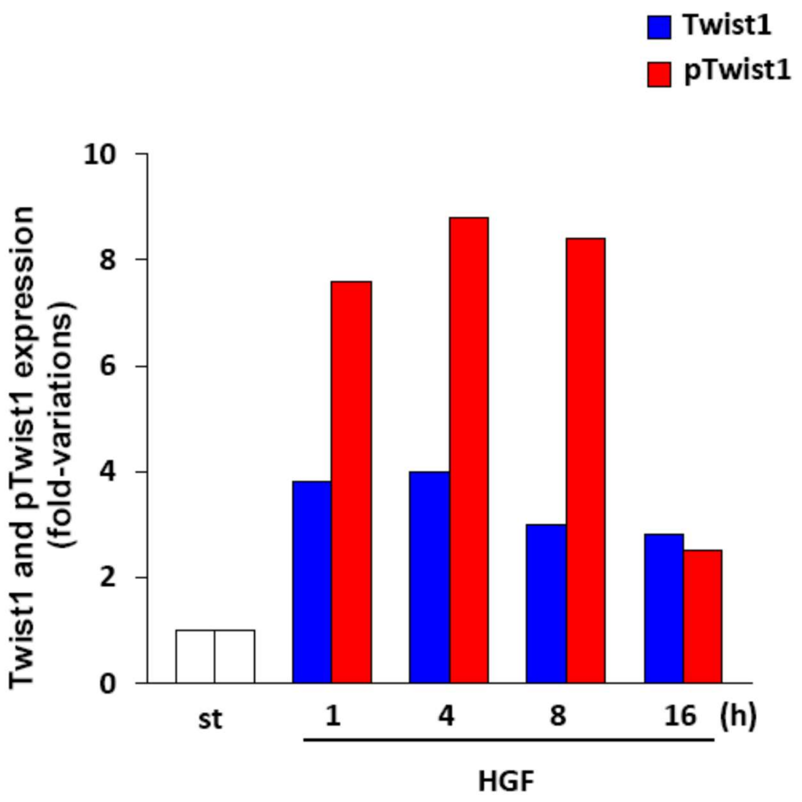

Supplementary Figure 2. Evaluation of Twist1 and phosphoTwist1 expression in nuclei of 1833 cells treated with HGF. Starved 1833 cells were exposed to 100 ng/ml HGF, and 50 μg of protein extracts from nuclei were analyzed by Western blot. Twist1 protein levels increased starting from 1 until 16 h from HGF exposure. Twist1 was strongly phosphorylated at these times, peaking between 1 and 8 h. The data indicated that the Twist1 transcription factor was active under HGF exposure.
